# Multidimensional analysis of road traffic noise and probable public health hazards in Barisal city corporation, Bangladesh

**DOI:** 10.1016/j.heliyon.2024.e35161

**Published:** 2024-07-24

**Authors:** Rifat Islam, Aliza Sultana, Md. Selim Reja, Ashraf Ali Seddique, Md. Rajib Hossain

**Affiliations:** a Department of Environmental Science & Disaster Management, Bangabandhu Sheikh Mujibur Rahman Science & Technology University, Gopalganj, 8100, Bangladesh; b Department of Environmental Science and Engineering, Jatiya Kabi Kazi Nazrul Islam University, Trishal, Mymensingh, Bangladesh

**Keywords:** Road Traffic Noise, Noise mapping, Human Health Hazards, Geographical information system, Spatial interpolation approach

## Abstract

Noise pollution is a major challenge in urban contexts all around the world. The study was designed to assess road traffic noise pollution with possible health effects on those living in the study region. The IDW spatial interpolation approach and an ArcGIS-based evaluation were used to map the recorded noise levels in the research region. The noise descriptors including Noise Climate (NC), Traffic Noise Index (TNI), Equivalent Noise Level (Leq), and Noise Pollution Level (NPL) were computed. The required information has been collected through a questionnaire survey and previously published documents. The study reveals that the current noise level is higher than the recommended national threshold at every location. According to the study, the Nathullabad region had the highest level of noise pollution (86.5 dBA), while the Kaunia Abasik area had the lowest level (67.8 dBA). Study findings also show that in the area context, the highest levels of noise pollution are found in commercial areas (82 dBA), followed by industrial areas (80.4 dBA),mixed areas (81.3 dBA), and residential areas (72.7 dBA). The lowest level is found in sensitive areas (72.5 dBA). Statistical analyses, including one-way ANOVA, Tukey HSD post-hoc and LSD post-hoc test results, showed that there was no statistically significant difference (*p* > 0.05) between the noise pollution levels (NPL) in the morning, noon, and evening shifts. The results showed that 32 % of respondents stated they felt disturbed while working, and 27% of respondents said it was somewhat sensitive for them. As the last step in minimizing noise pollution in the research area, 37 % of respondents reported enforcing the regulations, 31% suggested making hydraulic horns illegally, and 21 % suggested raising public awareness. This study may contribute to academic knowledge and assist decision-makers of government officials in formulating appropriate local strategies to deal with this grave environmental problem.

## 1 Introduction

An important environmental problem that affects urban dwellers is road traffic noise pollution. The word “Noise” comes from the Latin word nausea, which denotes seasickness [1]. The term “noise” is often used to describe an overly loud or disturbing sound that disturbs quiet. When the loudness reaches an unbearable level, it can be referred to as “noise pollution” in the atmosphere [1]. In the modern world, noise is recognized as one of the major environmental pollutants and serious health hazard which can come from a variety of sources [2]. Road traffic [3,4] railway traffic [5], construction industrial activities [6], crowded urban roads and miking [7] are the main sources of this type of noise exposure.

Worker stress levels and productivity may be affected by noise. It may also irritate and harm their hearing at high noise levels [8]. According to research results, noise levels above 60 dBA can influence a person's physical and mental health [9]. Noise is hazardous to public health [2,10,11]. Several pieces of research have revealed that noise has negative effects on people's health who reside in major City Corporation areas [4,12]. It can cause headaches, insomnia, psychiatric disorders, lack of focus, hearing loss, learning difficulties, stroke, and hypertension as well as lower quality of life [12]. At the same time, we have studied the noise pollution in the city corporation area. To uncover detailed information about Road Traffic Noise, and Probable Health Hazards on Adjacent Residents in this area.

GIS is now widely employed in research for various applications [11,13]. In some research, traffic noise maps are developed for the study area during day and night using various multidimensional analyses, GIS, and GPS [11,[13], [14], [15], [16], [17], [18], [19], [20]]. A more precise evaluation of noise pollution is made possible by the use of noise mapping [[16], [17], [18]]. The distribution of traffic noise is intuitively shown on the noise maps, which is useful for assessing traffic noise pollution. In some research [16,21], three to four noise maps depicting the morning, midday, evening, and night of the study region were shown, and the study area was separated into various area types for noise pollution evaluation. In certain studies, the study area's noise map is created using interpolation of monitoring data from noise monitoring stations or manually gathered data, and the traffic noise pollution is assessed using the equivalent sound pressure of day or night [17,22,23]. A useful tool for measuring noise in urban areas is noise mapping, which is a graphic representation of the noise level distribution in a specific area. Additionally, it helps in the representation of noise distributions in areas with a lot of land uses that are susceptible to noise. It helps with planning to decrease the effects of noise pollution [24,25].

Barisal City Corporation is a developing area, where the amount of noise pollution is increasing day by day because of an increasing number of vehicles and hydraulic horns. The study was carried out to quantify noise pollution, inspired by the study conducted in other city corporation areas of Bangladesh [26,27]. Therefore, this research must be carried out to monitor the level of road traffic noise from noise-making sources and potential health risks to the adjacent population in the study region. It might advance academic understanding and assist policymakers in formulating solutions to deal with this important environmental problem locally.

Several pieces of research work can be found using Multidimensional Analysis [13,28,29]. A recent study provided statistical evidence to support the potential health risk associated with road traffic noise pollution and issued a warning when it surpassed the level that is safe for human health [30]. Barisal City Corporation is a developing area. Day by day the number of vehicles here is increasing which is visible. No research on noise pollution has been organized in this area before. Therefore, we assume that the amount of noise pollution in the area will be high and people are already suffering. So, considering the above reasons we conducted the said new study. This study did not consider any medical devices to evaluate the health of Barisal City individuals who lived close to the noisy road. Additional research is required to validate the true health issue resulting from noise pollution. The research attempts to evaluate noise pollution in Barisal City Corporation by measuring noise pollution levels at several locations. It also provides an idea concerning the perception of the respondents toward noise pollution as well as their health condition together with the solutions for reducing noise pollution at traffic junctions in Barishal City Corporation, Bangladesh.

## 2 Materials and method

### 2.1 Framework of the study

This section presents the conceptual framework of the methodologies for the study.

**Figure:**
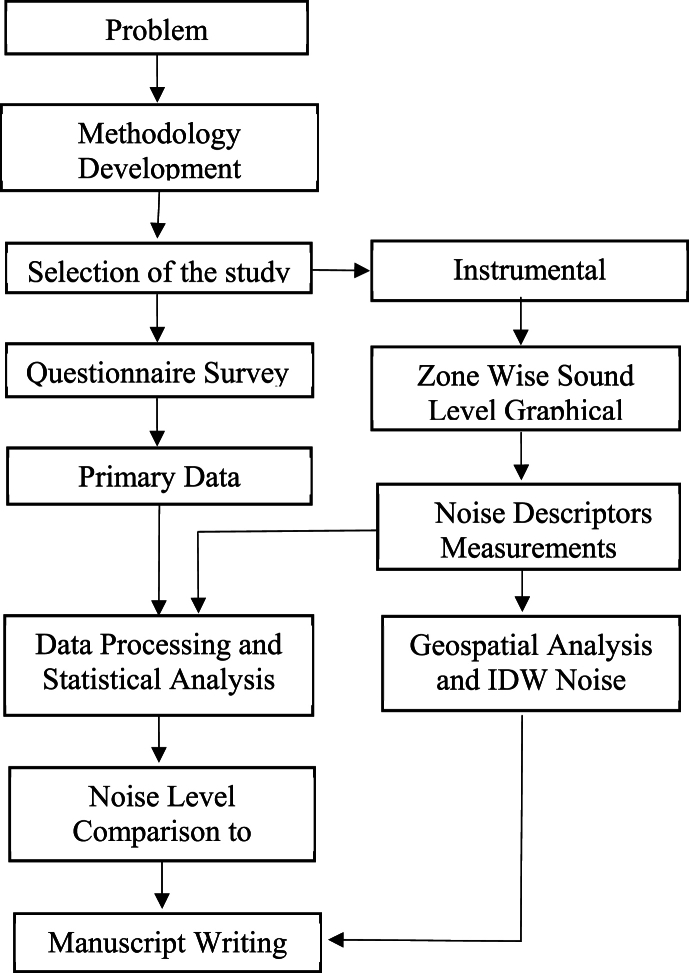

### 2.2 Study area

**Fig. 1:**
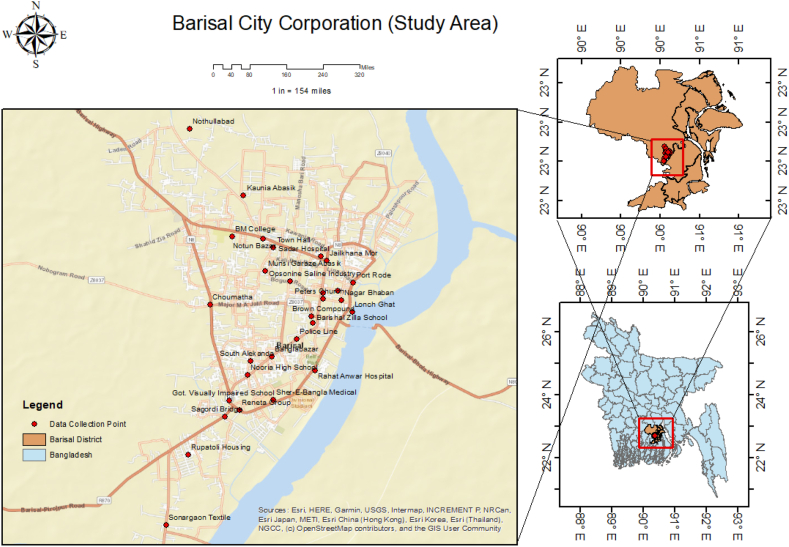
Barisal City Corporation (location point of the study area).

Due to traffic, recreational, religious, and other activities, it was discovered that the main roads and traffic intersections had become congested and noisy locations in the Barisal City Corporation. For this reason, 29 locations were randomly chosen for the study. Table 1 and Fig. 1 presents all the selected locations that fall in different zones.

**Table 1:** Geographical location of the study area.

Zones	Locations	Latitude	Longitude
SENSITIVE	Sadar hospital	22.7086	90.3703
	Bm college	22.7116	90.3567
	Peters church	22.70206	90.37062
	Jame koshai mosque	22.7033	90.3729
	Sher-e-bangla medical	22.6865135	90.3630105
	Rahat anwar hospital	22.6910177	90.3693543
	Barishal zilla school	22.6983706	90.3690594
	Nooria high school	22.69037897	90.35906317
	Got. Visually impaired school	22.686385	90.356225
COMMERCIAL	Notun bazar	22.7113	90.3614
	Bibir pukur par	22.70297599	90.37062407
	Banglabazar	22.69319857	90.362692
	Port rode	22.70458355	90.37528306
MIXED	Nothullabad	22.7282	90.3501
	Choumatha	22.70115463	90.35325556
	Town hall	22.71	90.363
	Nagar bhaban	22.7018	90.3734
	Lonch ghat	22.7	90.3751
	Sagordi bridge	22.68399241	90.35556571
	Jailkhana mor	22.70797682	90.37115753
	Police line	22.69584637	90.36658843
RESIDENTIAL	Rupatoli housing	22.67807002	90.34991736
	Kaunia abasik	22.71795202	90.3583882
	Brown compound	22.69936315	90.36884565
	South alekanda	22.69249472	90.35948925
	Munsi garaze abasik	22.70635679	90.36169403
INDUSTRIAL	Sonargaon textile	22.66727599	90.34645977
	Reneta group	22.68495814	90.35775909
	Opsonine saline industry	22.70472747	90.36558746

### 2.3 Sampling procedure

The primary data for this study were gathered using a probability selective sampling method based on a random selection of the research locations in each traffic intersection, a reconnaissance study was conducted to acquire precise data on the research region. Secondary data was acquired from a variety of sources such as various books and published articles. A structured questionnaire was used for the initial survey which was rather simple to read on a technical level Examining the health concerns associated with noise at traffic intersections can reveal their perception regarding how individuals feel about the issue. A simple random sampling process is used to collect the required data to perform the questionnaire survey. 400 respondents were chosen from different ages people including students, traffic police, street hawkers, shopkeepers, drivers, and passengers who were directly exposed to noise. The questionnaire asks about the respondents' racial and ethnic backgrounds, the main causes of noise pollution in those places, the effects of noise pollution on respondents' health, the many issues that noise pollution caused them, and potential solutions. It was conducted for three months (February, March, and April of 2023).

### 2.4 Data collection and materials

The IEC 651, type II standard-compliant precision grade Sound Level Meter DT-8850, with a low (35–100) dBA, high (65–130) dBA measuring range, and 0.1 dBA resolution, was the instrumentation used for the field measurements. The instrument has a 94 dBA built-in calibration check. The device was held pleasantly in the hand, with the microphone aimed at the possible source of noise at a height of about 1.5 m above the ground and more than 1m from the noise source, such as vehicles [26]. The apparatus was calibrated to A-weighting to generalize noise levels before doing measurements.

**Fig. 2:**
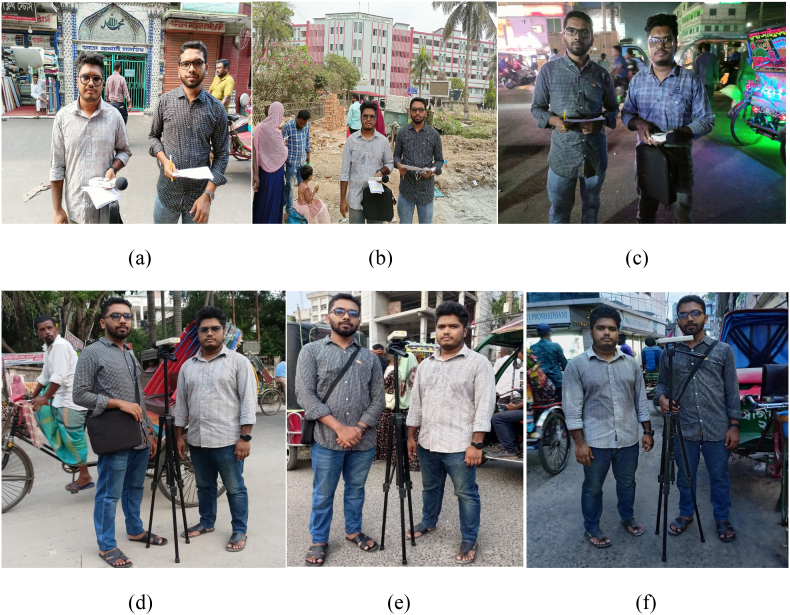
Monitoring times for noise pollution in three shifts: morning (a, d), noon (b, e), and evening (c, f).

The research area's longitude and latitude were measured using a Global Positioning System (GPS) device (Model: Garmin etrex-20). All the instruments comply with IEC (International Electrotechnical Commission) standards. The mapping of noise pollution in this research was done using Arc GIS 10.5 software. The questionnaire survey was conducted by Google form. The photographs taken during the research area's noise level monitoring at various shifts during the day are shown in Fig. 2(a, b, c, d, e, and f).

According to the Bangladesh Noise Pollution Control Rules, 2006 (7888 pages) [27], noise data were collected during the daytime. From three shifts throughout the daytime: morning (7:00 a.m.-10:10 a.m.), noon (12:00 p.m.-03:10 p.m.), and evening (05:00 p.m.-08:10 p.m.) the data were collected for various locations of the study area. Measurements were made under typical meteorological circumstances throughout working days at set intervals of 10 min [17].

### 2.5 Data processing, analysis, and interpretation

The study has been conducted employing multidimensional analysis [[23], [24]]. It helps to comprehend the present state of noise pollution in that area. Multiple descriptors were used to compute noise pollution levels in various locations in addition to GIS-based noise mapping. The statistical analysis explores the idea that noise pollution levels (NPL) vary depending on the morning, noon, and nighttime work shifts of the day and to pinpoint any noteworthy variations. As stated in the current objectives, all collected data were arranged, categorized, and reviewed before being processed for statistical analysis utilizing IBM SPSS Statistics 26 and Microsoft Excel of the Office 2021 version.

To calculate the average noise pressure level Lp dBA, and equivalent continuous equal energy level, Leq dBA, equations (1), (2) were utilized [28]. Lp dBA represents the averge sound pressure level and is a measure of the intensity of sound in each environment. It is commonly used to quantify and compare the loudness of different sounds, including environmental noise [26,29].

(1) $$Lp=10\;\log\sum 10\left(\frac{li}{10}+\frac{lk}{10}\right)$$

NPL dBA refers to the overall level of noise pollution in a specific area. It represents the average or equivalent continuous noise level that has been present at a particular location over a given period. NPL is a fundamental parameter used to evaluate the general noise environment in an area and assess its potential impact on human health and well-being [29].

(2) $$NPL=L_{50}+\left(L_{10}-L_{90}\right)+\frac{{\left(L_{10}-L_{90}\right)}^{2}}{60}$$

Leq is a time-weighted average of sound levels over a specific period, typically measured in decibels dBA, which is utilized in equation (3). It represents the steady, continuous noise level that would produce the same energy as the fluctuating or intermittent noise over the same period. Leq is commonly used to assess and compare noise exposure levels over an extended time frame. It is a key descriptor of discomfort caused by any type of fluctuating noise level, such as the noise from vehicles passing by, over a particular period [29].

(3) $$Leq=L_{50}+\left[\frac{{\left({L_{10}-L}_{90}\right)}^{2}}{56}\right]$$

Traffic noise unpleasantness is indicated by the Noise Climate (NC) dBA [23]. The difference between the peak noise and background noise level in each area increases with the magnitude of noise climate (NC). The range of sound levels that change over a particular period is known as the noise climate, and it is calculated using the relation shown in equation (4) [29].

(4) $$NC=\left(L_{10}-L_{90}\right)$$

Traffic noise variation is shown by the traffic noise index (TNI) dBA, which gauges human annoyance behavior. It shows the general changes in noise levels over time as well as the disarray of traffic noise levels. Equation (5) can be used to determine the Traffic Noise Index (TNI) dBA [12,29].

(5) $$TNI=4\times \left(L_{10}-L_{90}\right)+\left(L_{90}-30\right)$$

All the column charts show the indicated error bars with respect to the standard deviation of the NPL values [4].

#### 2.5.1 Noise mapping

Noise mapping is one of the most contemporary methods of measuring noise levels. Using ArcGIS 10.5 software [30], the Barisal City Corporation noise map was produced for this investigation. The software employs the Inverse Distance Weighting (IDW) interpolation technique. IDW produces good results when multiple elevation points are distributed uniformly across a region. When using the IDW Interpolation approach [23], all data collected from noise sources and the distances between them are taken into consideration. The three times of the day's acoustics in the area were measured using this technique to determine their geographical distribution and range [12,26]. The method chosen for the measurements was determined by examining many similar studies in scientific literature. When these studies are investigated, it is seen that similar measurement methods and measurement periods are used [3,[16], [17], [18], [19], [20],[31], [32], [33], [34]].

### 2.6 Statistical analysis

The homogeneity of variance (Levene's Test) and normality (Kolmogorov-Smirnov^a^ and Shapiro-Wilk tests) assumptions were applied. To determine whether there is a significant difference in noise pollution levels (NPL) between the morning, noon, and evening shifts of the day, a one-way analysis of variance (ANOVA) with Tukey HSD and LSD post-hoc test was carried out in this study. The morning, noon, and evening shifts of the day were the fixed factors for the ANOVA, and the noise pollution level (NPL) was the dependent variable. As a result, the alternative hypothesis (H_1_) suggests that the effects of the NPL are not equivalent for at least one of the two shifts, whereas the null hypothesis (H_0_) suggests that the effects of the NPL are similar for all day shifts. The null hypothesis is considered to be retained if the produced *p* value is greater than the significance level (0.05) [35,36]. To confirm the validity and the visualization of the public response questionnaire, a reliability test was also performed.

## 3 Results

### 3.1 Zone-specific noise measurement

**Fig. 3:**
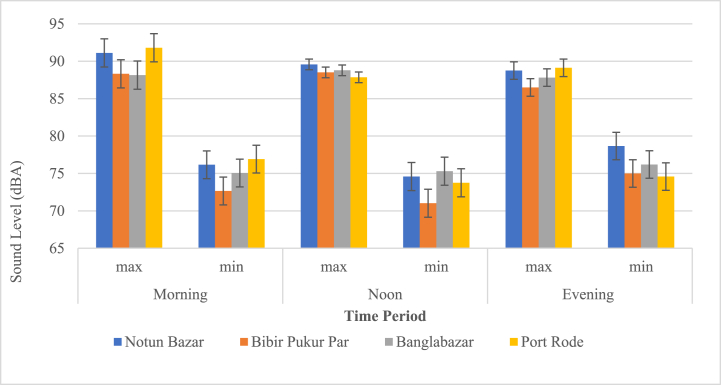
Daytime sound pressure levels in the Commercial zone.

In the commercial zone of the Barisal City Corporation Port Road area, it can be shown that the maximum noise level recorded in the morning (91.80 dBA). Additionally, the Notun Bazar region had also the highest minimum noise level (78.7 dBA) in the evening (Fig. 3).

**Fig. 4:**
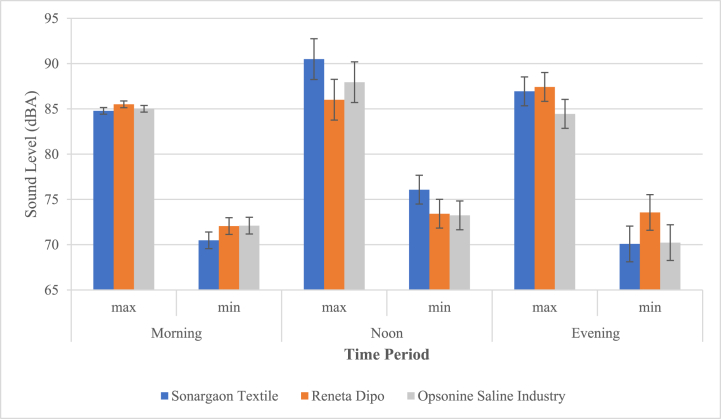
Daytime sound pressure levels in the Industrial zone.

Fig. 4 shows that the Sonargaon Textile industrial zone recorded the highest maximum noise level (90.5 dBA) during the noon shift, whereas the evening noise level was found to be the lowest (70 dBA) on a minimum scale.

**Fig. 5:**
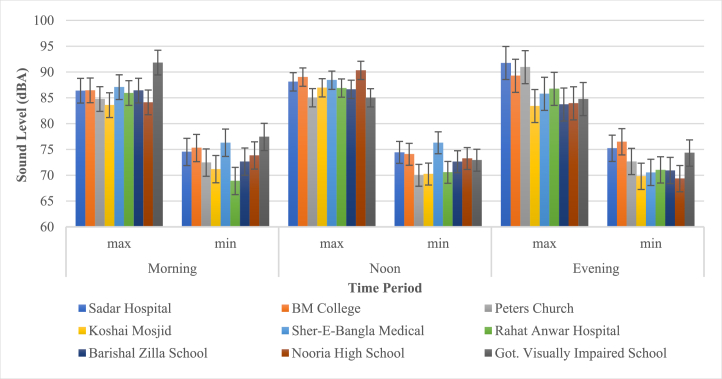
Daytime sound pressure levels in the Sensitive zone.

From Fig. 5, the sensitive zone of the Barisal City Corporation Sadar Hospital area had a maximum noise level of 91.7 dBA during the evening. Additionally, the Sher-E-Bangla Medical area had the highest minimum noise level in the morning 76.29 dBA. whereas the Rahat Anwar Hospital area had the lowest noise level (70 dBA) in the evening on a minimum scale (Fig. 5).

**Fig. 6:**
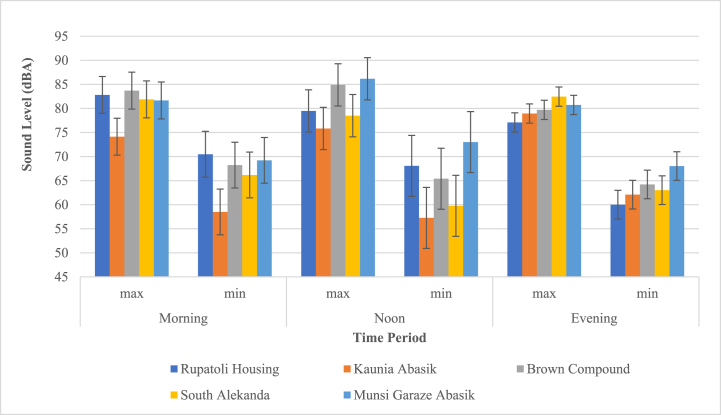
Daytime sound pressure levels in the Residential zone.

The maximum noise level recorded at noon for the Brown compound region (Residential zone) is 86.2 dBA. The Kaunia Abasik area had the lowest noise level (57.3 dBA) at noon. (Fig. 6).

**Fig. 7:**
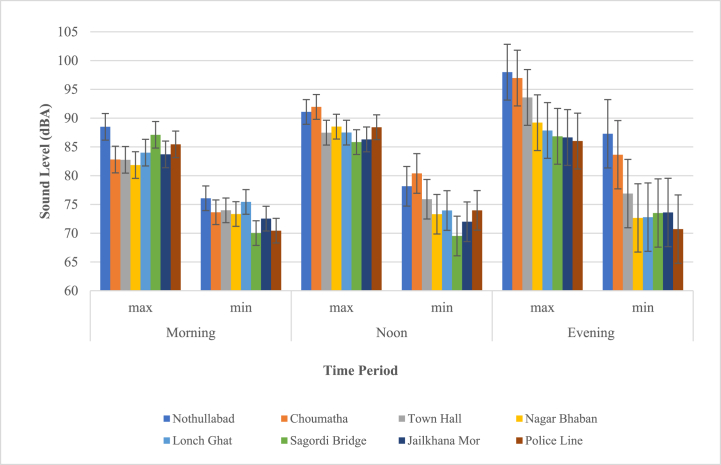
Daytime sound pressure levels in the Mixed zone.

Fig. 7 displays that the maximum noise level recorded in the evening for the mixed zone of Barisal City Corporation (Notullabadh region) is 97.98 dBA. The lowest noise level was 69.5 dBA in the Sagardi Bridge area at noon.

### 3.2 Zone-wise comparison of noise level with acceptable limits

**Fig. 8:**
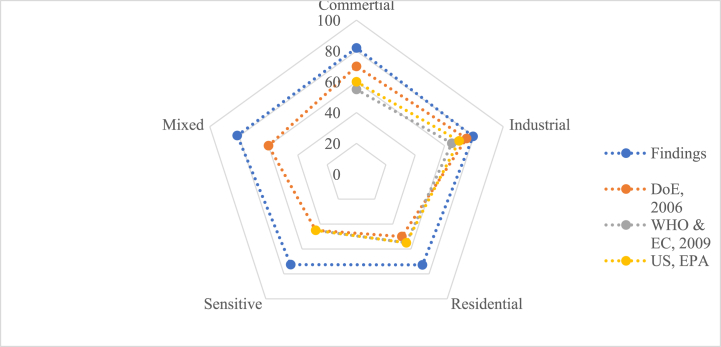
Comparison of sound pressure level with standard values by zone.

The sensitive areas had the lowest average noise level (72.50 dBA), which was over the permitted limit, while the commercial areas had the highest average noise level (81.92 dBA) (Fig. 8).

The NPL and Leq in commercial areas in the research region ranged from 92.2 to 112.0 dBA and 81.7 to 89.2 dBA, respectively. The Bangla bazar zone recorded the lowest NPL (92.2 dBA) at noon, whereas the port road region recorded the highest NPL (112.0 dBA) in the morning (Table 2). The Bangla bazar zone had the lowest NPL (92.2 dBA) around noon, while the port road region recorded the highest NPL (112.0 dBA). (Table 2). The highest average daytime Leq and NPL in the sensitive zone were discovered at BM College (84.57 dBA) and Sher-E-Bangla Medical College (98.46 dBA). In the commercial zone, Notun Bazar (86.5 dBA) and Port Road (104.06 dBA) had the highest average daytime Leq and NPL respectively. The highest average daytime Leq (89.96 dBA) and NPL (109.03 dBA) in the mixed zone were both discovered at Nothullabad The highest average daytime Leq (89.96 dBA) and NPL (109.03 dBA) among the mixed zones were discovered at Nothullabad. Monsi Garaze had the highest average daytime Leq (79.76 dBA) and NPL (97.3 dBA) among the residential zones. Reneta Group (83.4 dBA) and Zailkhana Mor (99.53 dBA) among the industrial zones had the highest average daytime Leq and NPL (Table 2).

**Table 2:** Calculated day-time Noise level parameters for three shifts at 29 locations in Barisal City corporation including L_90_ dBA, L_50_ dBA, L_10_ dBA, Equivalent noise level (Leq), Noise Climate (NC) Noise Pollution level (NPL) and Traffic Noise Index (TNI).

Location	Parameters																				
	L_90_ dBA			L_50_ dBA			L_10_ dBA			Leq dBA			NPL dBA			Noise Climate			TNI		
	Morning	Noon	Evening	Morning	Noon	Evening	Morning	Noon	Evening	Morning	Noon	Evening	Morning	Noon	Evening	Morning	Noon	Evening	Morning	Noon	Evening
**Sadar Hospital**	74	76.9	76.7	80.2	80.3	85.3	87.5	89.8	88.5	82.3	83.8	85.2	96.7	96	99.4	13.5	12.9	11.8	78	98.5	93.9
**BM College**	76.6	75.6	77.9	81.9	80.7	82.7	85.7	90.4	89.7	82.2	84.1	87.4	92.4	99.2	96.8	9.1	14.8	11.8	42.8	104.8	95.1
**Peter Church**	72.2	72.7	76	78.2	78.8	82.3	85.4	80.6	87.5	80.7	78.8	84	94.3	87.7	96	13.2	7.9	11.5	75.6	74.3	92
**Koshai Mosjid**	69.4	72.3	71.4	78	77.8	76	87.1	86.4	83.2	82.2	81	79	101	95.2	90.1	17.7	14.1	11.8	112	98.7	88.6
**Sher-E-Bangla Medical**	75.8	77	72.8	80.1	81.7	78	92.3	92.5	85.3	85.6	87.1	80.2	101.1	101.2	93.1	16.5	15.5	12.5	102	109	92.8
**Rahat Anwar hospital**	70.9	71.9	74.7	77.9	79.3	79	83.5	85.8	83.1	78.9	80.7	80.1	93.1	96.4	88.6	12.6	13.9	8.4	70.8	97.5	78.3
**Barisal Zila School**	74.3	74.5	73.1	79.1	78	76.7	85	89	81.5	81	82.9	77.8	91.7	96	86.3	10.7	14.5	8.4	55.6	102.5	76.7
**Nooria High School**	75.2	76.1	71.3	79	82.4	77.4	84.7	88.8	82.2	80.2	83.9	78.1	90	97.8	90.3	9.5	12.7	10.9	46	96.9	84.9
**Govt.Visually impaired School**	80.7	72.15	69.1	83.5	77.75	82.2	88.8	86.1	86.5	86.2	81.1	82.7	92.7	94.9	104.6	8.1	13.95	17.4	34.8	97.95	108.7
**Notun Bazar**	75.9	76.8	76.7	82.2	83.6	82.6	94	86.6	91	89.2	83.3	87	106	95	100	18.1	9.8	14.3	115	86	104
**Bibir Pukur Par**	71.4	73.8	72.5	82	80	82	87.4	86.3	87.3	83.6	81.7	83	102	95.1	101	16	12.5	14.8	98	93.8	102
**Bangla Bazar**	76	77.8	76.5	87.7	80.5	81	85.6	87.8	88.5	83	83.9	84.3	98.8	92.2	95.4	9.6	10	12	46.8	87.8	94.5
**Port Road**	71	74.5	74.6	85.2	80.3	81	90.8	91.3	89	86.8	84.3	84.6	112	102	98.9	19.8	16.8	14.4	128	112	102
**Nothullabad**	70.8	79.4	84,4	81.2	85.4	95	94.2	89.5	101	87.8	86.1	96	114	97.2	116	23.4	10.1	16.6	157	89.8	121
**Choumatha**	70.4	81.6	83	78.4	85.4	90.6	82.7	93	96.7	80.3	90	92.7	93.2	99	107	12.3	11.4	13.7	68.4	97.2	108
**Town Hall**	72.7	78.6	77.2	79.4	81	85.5	83.2	86.6	93.8	80	82.6	88.3	91.7	90.1	107	10.5	8	16.6	54	80.6	114
**Nagar Bhaban**	75.5	73.6	76	77.7	81.4	80.5	80.4	88.1	87.3	78.1	83.2	82.8	83	99.4	93.9	4.9	14.5	11.3	9.2	102	91.2
**Rupatoli Housing**	68.5	66.3	63.7	75.7	73.7	68	86.4	82.1	76.4	81.6	76.5	70.8	98.9	93.7	83.4	17.9	15.8	12.7	113	99.5	84.5
**Kaunia Abasik**	58	62.6	63.4	62.8	66	69	81.1	71	81.4	74.9	67.8	75.5	94.8	75.6	92.4	23.1	8.4	18	155	66.2	105
**Brand Compound**	68.6	68.4	67.3	76	74.4	71	83.5	85.4	78	78.6	79	73.9	94.6	96.2	83.7	14.9	17	10.8	89.2	106	80.3
**South Alekanda**	65.8	63.2	68.1	73.4	69.3	72.6	82	74.4	78	78.3	70.5	74.1	94	82.6	84.1	16.2	11.2	9.9	99.6	78	77.7
**Monsi Garaze**	69.4	73.7	63.1	76	79.5	75.5	81.3	86.6	85.6	77.5	82.2	79.6	90.3	95.2	106	11.9	12.9	22.5	65.2	95.3	123
**Sonargaon Textile**	72.2	78.3	72	76.5	81	78.6	86.6	95	83.4	81.5	87.8	80.4	94.4	102	92.2	14.4	16.7	11.4	85.2	115	87.6
**Reneta Group**	73.7	73.5	73.7	77.2	79.5	79.6	89	84.3	87.1	84.9	83	82.3	96.4	92.2	96	15.3	10.8	13.4	92.4	86.7	97.3
**Opsonine Salyine Industry**	70.4	74.8	70	78.7	80.8	78.6	86.9	89.8	83.4	82.2	83.3	79.7	99.7	99.6	95	16.5	15	13.4	102	104.8	93.6
**Lonch Ghat**	73.7	71.6	76.2	79.4	80.1	80	87	89.5	89.1	82.6	84	83.4	95.6	103	95.7	13.3	17.9	12.9	76.4	113	97.8
**Zailkhana Mor**	68.6	73.2	72	78.4	78.3	78	87	86.3	90.3	81.6	81.6	84	102	94.3	102	18.4	13.1	18.3	117	95.6	115
**Sagordi Bridge**	69.2	72.1	77	79.5	78.4	81.3	83.6	86.4	86	80	80.4	81.7	97.4	96.1	91.7	14.4	14.3	9	85.2	99.3	83
**Police Line**	73.1	76.5	69	77.5	81.1	79	82	87.3	88.4	79.1	82.8	81.6	87.7	93.8	105	8.9	10.8	19.4	41.2	89.7	117

According to calculated Leq, the categorization obtained in decline order is as follows: commercial zone > mixed zone > industrial zone > sensitive zone > residential zone. Additionally, for NPL the sequence can be observed for daytime: commercial zone > industrial zone > mixed zone > sensitive zone > residential zone Table 2.

### 3.3 Measurement of daytime community noise level

Noise level parameters reported in Table 2 display the daytime community noise descriptors levels L_10_ dBA, L_50_ dBA, and L_90_ dBA. Here L_10_ represents the noise level that is exceeded 10 % of the time. In other words, 90 % of the time, the noise level is lower than the L_10_ value. L_10_ is often associated with short-term, sporadic noise events that may occur relatively frequently. L_50_ represents the noise level that is exceeded 50 % of the time, or the median noise level. It indicates the point at which half of the noise measurements are below the L_50_ value and half are above it. L_50_ provides a good representation of the typical noise conditions in the area. L_90_ represents the noise level that is exceeded 90 % of the time. It indicates the higher range of noise levels that occur less frequently. L_90_ is often associated with more constant or continuous noise sources that may not occur as frequently as those represented by L_10_ [37].

Table 2 indicates a comprehensive overview of noise descriptors at specific sites within Barisal City Corporation, with data collected during morning, noon, and evening shifts. These sites encompass a diverse range of establishments, including hospitals, colleges, mosques, schools, and other public areas. Through a thorough analysis of the information presented in Table 2, a clear understanding of the noise characteristics and fluctuations in different regions of Barisal City at various times of the day emerges. This critical data enables the assessment of noise pollution levels, the identification of potential noise hotspots, and the formulation of effective noise control strategies to enhance the well-being and comfort of the city's residents.

### 3.4 Noise mapping

**Fig. 9:**
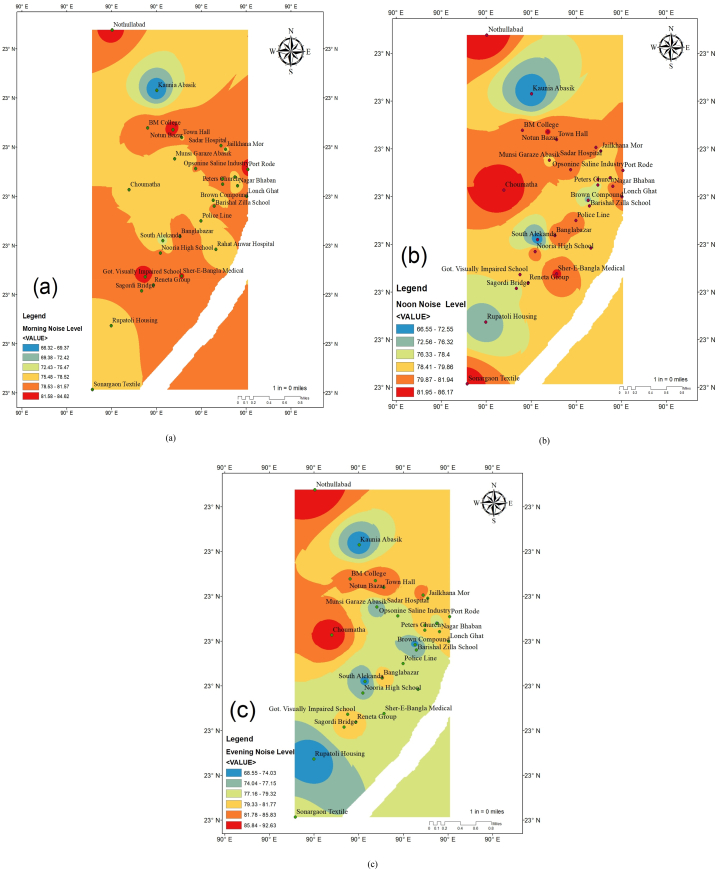
Mapping of the spatial variation in noise levels in the morning (a), at noon (b), and in the evening (c) in Barisal City Corporation.

Noise mapping which shows the distribution of sound levels in a specific area graphically, is an effective way to measure noise in City Corporation environments. Additionally, the spatial variation mapping of noise levels for the morning, afternoon, and evening hours in Barisal City Corporation is depicted in Fig. 9 (a, b, c) respectively.

There are six different colors on the map in Fig. 9 (a), each one denoting a different level of noise pollution (NPL) in the morning shift. Only the Kaunia residential area's noise levels ranged from 66.32 to 69.37 (blue color). The light green hue denotes noise levels between 72.43 and 75.47, while the green color represents noise levels between 69.38 and 72.42. Within this pale green spectrum is where the South Alek residential area is located. Sonargoan Textile, Rupatoli Housing, Choumatha, Police Line, Rahat Anwar Hospital, Brown Compound, Munsi Garaze Abask, Bibir Pukur Par, Nagar Bhavan, Town Hall, and Jailkhana Mor are just a few of the notable locations that fall within the range between 75.48 and 78.52 (yellow color). On the map, noise levels ranging from 78.53 to 81.57 are depicted in orange color. This orange spectrum includes coverage for ten prominent locations including Sagordi Bridge, Reneta Group, Nooria High School, Bangla Bazaar, Barisal Zilla School, Lonch Ghat, Peter Church, Jame Kosai Mosque, Opsonine Saline Industry, Sadar Hospital, and BM College. The final sign of a high sound level is a red star, which appears when the noise level is between 81.58 and 84.62 dB. Govt. Visually Impaired School, Sher-E-Bangla Medical, Port Road, Notun Bazar, and Notullabad are among the locations served by this range.

Fig. 9 (b) shows spatial variation in noise levels at noon shift (b) in the study area. The map shows different noise pollution levels (NPL) as distinct colors. The lowest noise level, which ranges from 66.5 to 72.55 dBA, is indicated by blue color. The Kaunia residential area and the South Alekanda area are included in this range. The green color designates areas of Rupatali Housing and Bround Compound Road where noise levels range from 72.56 to 76.32 dBA. Sagordi Bridge and Peter's Church are represented by a range of 76.33–78.4 dBA in the light green color. The yellow color on the map denotes decibel ranges of 78.41–79.86. Renata Group, Govt. Visually Impaired Schools, Rahat Anwar Hospital, Barisal Zilla School, Bibir Pukur Par, Jeilkhana Mor, and Munsi Garaze Abasik are a few significant localities in this range. Sonargaon Textile, Sher-E Bangla Medical, Chowmatha, Notun Bazar, and Nathullabad are further areas with comparable noise levels.

The map's orange tint represents noise levels between 79.87 dBA and 81.94 dBA. Notable areas within this range include Bangla Bazar, Police Line, Lonch Ghat, Nagar Bhaban, Port Road, Opsonine Saline Industry, Sadar Hospital, Town Hall, and BM College. Finally, Sonargaon Textile, Sher-E Bangla Medical, Choumatha, Notun Bazar, and Nothullabad all fall inside the red color band, which denotes the maximum noise intensity.

The map in Fig. 9 (c) displays six colors to show different noise pollution levels (NPL). Rupatoli Housing, South Alekanda, Brown Compound, and Kaunia Abassik are all included in the blue (68.55–74.03 dBA) zone. Munsi Garaze is on the green (74.04–77.15 dBA). Sher-E-Bangla Medical, Rahat Anwar Hospital, Police Line, Bibir Pukur Par, and Opsonine Saline are all in the light green (77.1–79.32 dBA) zone. Sagordi Bridge, Reneta, Govt. Visually Impaired Schools, Banglabazar, Lonch Ghat, Nagar Bhaban, Peter's Church, Port Road, and Jailkhana Mor are all covered in yellow (79.33–81.77 dBA) color range. Orange (81.78–85.83 dBA) has Sadar Hospital, Town Hall, Notun Bazar, and BM College are included in Orange (81.78–85.83 dBA) color range. Chumatha and Nothullabad fall inside the red hue spectrum, which represents the loudest noise.

A one-way between-group analysis of variance (ANOVA) was employed to examine the effect of noise pollution level (NPL) on different daytime. Examining the results of the Shapiro-Wilk and Kolmogorov-Smirnova tests revealed that, the assumption of normality for the dependent variable (NPL) was not violated for morning and evening but for noon it was violated. Since the Levene statistic (*f* (2, 84) = 2.018, *p* = 0.139) was non-significant, thus the assumption test of Homogeneity of variances can be assumed.

The ANOVA results (Table 3) show that for the three shifts (morning, noon, and evening), there was no statistically significant difference (p > 0.05) in the NPL values: F (2, 84) = 0.340, p = 00.713 > 0.05. Using eta squared, the effect size (0.00803) was determined.

**Table 3:** One-way ANOVA test of NPL in different shifts of daytime.

ANOVA test					
Categories	Sum of Squares	*df*	Mean Square Value	*f*	*P*
Between Groups	31.236	2	15.618	0.340	0.713
Within Groups	3858.343	84	45.933		
Total	3889.579	86			

The post-hoc comparisons using the Tukey HSD test and LSD test results confirm the ANOVA findings. Tukey HSD test and LSD test indicated that the mean scores for morning, noon, and evening shifts did not differ significantly from each other (Table 4).

**Table 4:** Tukey and LSD posthoc test of NPL in different shifts of daytime on working and holidays.

Tukey HSD POST-HOC TEST			
(I) Shifts	(J) Shifts	Mean Difference (I-J)	Significant level
Morning	Noon	1.379	0.719
	Evening	0.255	0.989
Noon	Morning	−1.379	0.719
	Evening	−1.124	0.803
Evening	Morning	−0.255	0.989
	Noon	1.124	0.803
**LSD POSTHOC TEST**			
(I) Shifts	(J) Shifts	Mean Difference (I-J)	Significant level
Morning	Noon	1.379	0.441
	Evening	0.255	0.886
Noon	Morning	−1.379	0.441
	Evening	−1.124	0.529
Evening	Morning	−0.255	0.886
	Noon	1.124	0.529

## 4 Public perception

**Fig. 10:**
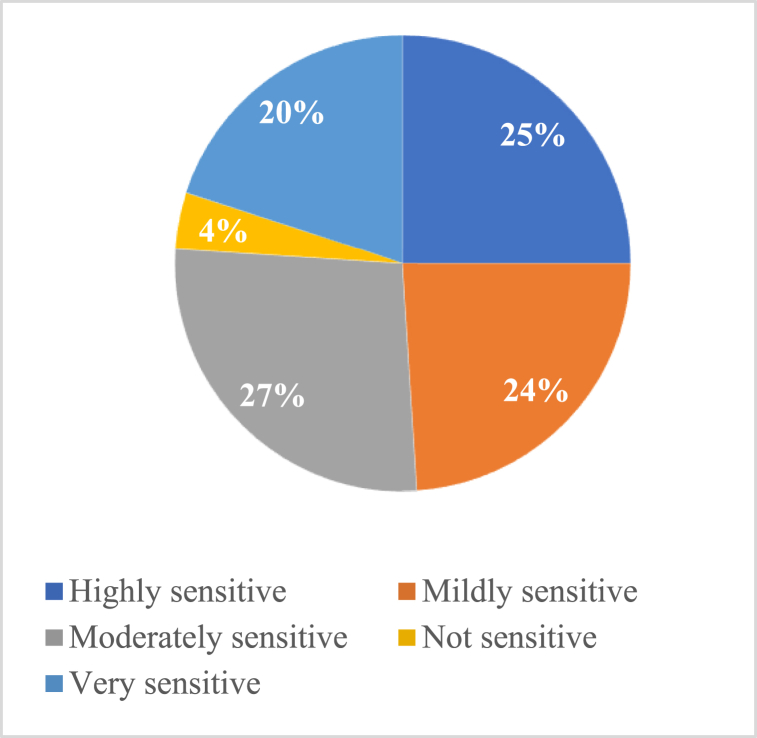
General noise pollution level status.

**Fig. 11:**
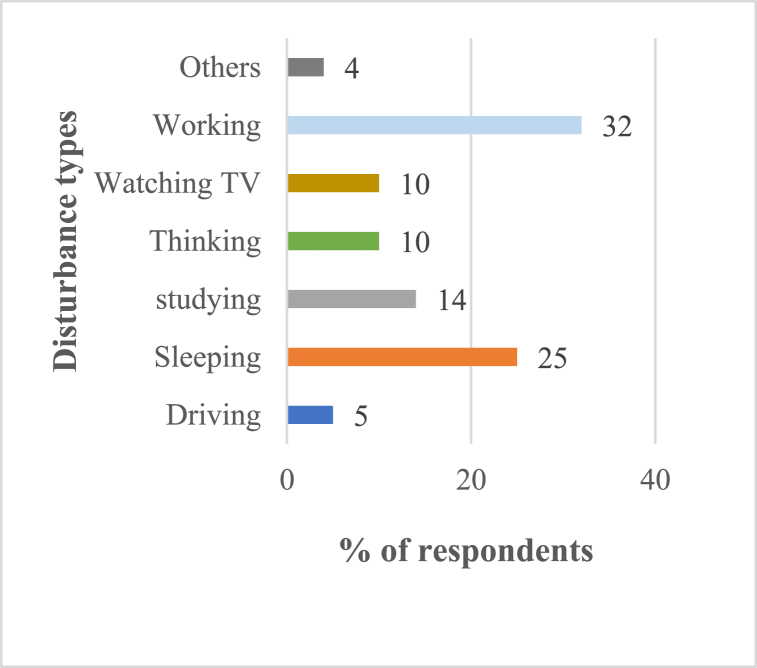
Effects of noise pollution on individual activities.

Fig. 10, Fig. 11 display the general noise pollution level status and the effect of it on the respondents. The findings of reliability test are displayed in Table 5, Table 6. 4 % of the respondents indicated that the noise pollution in the Barisal City Corporation region was not sensitive, and 27 % reported that it was moderately sensitive. According to the research, noise pollution had the greatest impact on people's daily activities that were related to their daily work, followed by sleeping, education, watching television, thinking, etc Fig. 10, Fig. 11.

**Table 5:** Reliability Statistics of the public responses.

Cronbach's Alpha	Number of Items
0.90	4

**Table 6:** Item Statistics of the public responses.

Public Perceptions	Mean	Std. Deviation
General noise pollution level status	2.7000	1.41778
Effects of Noise Pollution on Individual Activities	3.2100	1.89787
Nonauditory health impact of noise pollution	2.4700	1.30620
Action measures for controlling noise pollution	2.0500	0.86894

**Fig. 12:**
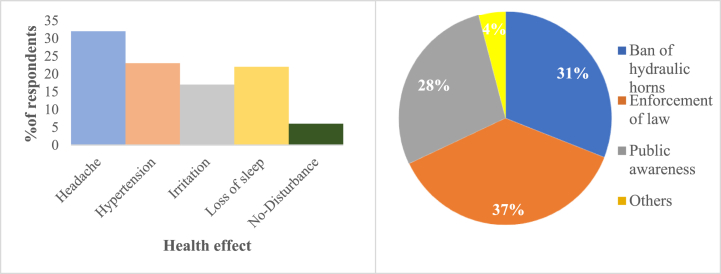
Non-auditory health impact of noise pollution and Action measures for controlling noise pollution.

Headaches, hypertension, Sleeping, and irritation were the effects of noise pollution on people that occur most frequently (Fig. 12).

When asked what actions should be taken to reduce noise pollution the majority (37 %) of respondents said that the law should be enforced, then those hydraulic horns should be banned (31 %) and finally that public awareness should be increased (28 %) (Fig. 12).

## 5 Discussion

The findings of this study corroborate existing literature on the significance and methodology of noise mapping in urban environments. Noise pollution is a serious problem in urban settings, including the Barisal City Corporation area. The analysis shows that every zone's current daytime noise level exceeded the permissible or acceptable limits specified by DoE, WHO, EC, and US, EPA (Fig. 8) [[36], [37], [38]]. Numerous studies were conducted in Bangladesh to evaluate noise levels and their impacts on human health, and it was discovered that the noise level exceeded the generally accepted permitted noise level [4,[39], [40], [41], [42], [43]]. The study reveals that the present noise level in all the locations exceeds the prescribed limit. The results show that the current noise levels in every place are higher than the permitted level for Bangladesh.

The population in this area is subjected to a high noise level, which is mostly brought on by traffic. In many cities in Bangladesh, the values of various noise parameters L_10_, L_90_, L_50_, NC, TNI, Leq, and NPL also increased [43,44]. This study also identified that at each location, all these values increased. This study also depicts that all these values became high at each location and the noise pollution level (Lnp) varied between 75.6 dB(A) to 116 dB(A) (Table 2). Compared to other zones, the commercial zone has a considerably greater noise level. Numerous studies have supported this view. The most problematic zone in Rajshahi, Bangladesh, was the commercial area, where the greatest noise level was 107.3 dBA [17]. According to different research conducted in Bangladesh obtained similar type results Joydebpur Junction had the greatest average noise level (100.9 dBA), while the Bangabandhu Satellite Ground Station had the lowest average noise level (47.5 dBA) [18].

These findings align with the literature that emphasizes the role of traffic, commercial activities, and urban infrastructure in elevating noise levels in city environments. The study highlights those areas with dense traffic and commercial activities, such as Sagordi Bridge, Reneta Group, and major medical facilities, consistently exhibit higher noise levels throughout the day. This is consistent with the observations in literature that noise levels vary with location and time of day due to traffic volume, vehicle horns, and other urban activities.

Noise levels are also above the permissible limits prescribed by the WHO. In a study in Chandigarh, India, up to 80 dB noise level was measured at the emergency and around. About 74 % of the total hospital patients said that they have irritation due to loud noise, 40 % reported a headache, 29 % reported a loss of sleep and 8 % reported hypertension [[45], [46], [47], [48], [49]]. Numerous health problems, including cardiovascular disease, hypertension, ear problems, and sleeping disorders, can be brought on by excessive noise in a community [9,50,51]. Long-term noise exposure may have detrimental health impacts, such as irritation, sleep difficulties, learning impairment, cardiovascular disorders, hearing loss, and tinnitus [27,52]. World Health Organization (WHO) states that noise levels of 60 dBA and 100 dBA can separately result in temporary deafness and full defect. WHO also reported that more than 5 % of people worldwide experience hearing impairment. This study also found several negative impacts on human health, including headaches, hypertension, lack of sleep, and irritability according to the perception of the respondents. This research report that Barisal City Corporation is becoming more and more polluted by noise. It is also advised that to lower the risk of noise pollution, people should be made more aware of its effects, and government agencies should put in place efficient noise control measures. The health implications of noise pollution, well-documented in the literature, are also evident in the study's findings. Excessive noise levels, particularly in areas with critical infrastructure like hospitals and schools, pose significant risks to the cardiovascular health, hearing, and overall well-being of residents [[53], [54], [55], [56], [57], [58]]. This study reinforces the need for targeted noise mitigation strategies in high-risk areas to protect public health, aligning with global standards and recommendations such as those from the U.S. Department of Housing and Urban Development (HUD) [[59], [60], [61], [62], [63]].

Noise mapping, as established, is a powerful tool for visualizing and assessing noise pollution levels across different areas of a city [64,65]. This study's detailed noise maps for Barisal City Corporation during morning, afternoon, and evening shifts vividly illustrate the spatial distribution of noise pollution, facilitating a deeper understanding of how noise levels fluctuate throughout the day in various city sectors. The morning noise map (Fig. 9a) reveals that the Kaunia residential area experiences the lowest noise levels (66.32–69.37 dBA), while the highest levels are found in areas such as Govt. Visually Impaired School and Sher-E-Bangla Medical, where noise levels range from 81.58 to 84.62 dBA. The afternoon noise map (Fig. 9b) shows a shift in noise pollution distribution, with the lowest levels (66.5–72.55 dBA) still in residential areas but the highest levels (79.87–81.94 dBA) spreading to commercial zones such as Bangla Bazar and Lonch Ghat. The evening noise map (Fig. 9c) indicates a slight increase in overall noise levels, with the lowest levels (68.55–74.03 dBA) still in residential zones and the highest levels (81.78–85.83 dBA) now covering critical areas like Sadar Hospital and BM College.

The European Parliament's Directive 2002/49/EC mandates the creation of noise maps for cities with populations exceeding 250,000 to manage and mitigate environmental noise pollution [66]. Although Barisal City Corporation has a smaller population, the methodology and findings of this study underscore the importance of such measures for smaller urban centers as well. The use of ArcGIS 10.8 software with the Inverse Distance Weighting (IDW) interpolation method provided reliable noise maps, supporting literature that endorses IDW for its accuracy in handling large and uniformly distributed data points [16,17]. This study's noise mapping of Barisal City Corporation provides a clear graphical representation of noise pollution distribution, highlighting critical areas needing intervention. By correlating these findings with established literature, it becomes evident that strategic urban planning and noise management policies are crucial for mitigating the adverse effects of noise pollution on urban populations. This research contributes to the broader understanding of urban noise pollution and underscores the need for continuous monitoring and effective policy implementation to enhance the quality of urban living environments.

## 6 Conclusion

This study demonstrated that noise pollution is a substantial issue in Barisal City Corporation.

According to the DoE, WHO, and US EPA, noise pollution is exceedingly generally considered safe levels in all zones (commercial, mixed, industrial, residential, and sensitive), endangering the health and well-being of the local population. ArcGIS-based evaluation and the IDW spatial interpolation technique display the greater noise pollution level in each site. According to the study, the Nathullabad region had the highest level of noise pollution (86.5 dBA), while the Kaunia Abasik area had the lowest level (67.8 dBA). The respondents verbally suggest that action be taken to lessen noise pollution in the city and to increase public awareness of the problem. he recommended mitigating strategies, which include education, administrative actions, and law enforcement, are all crucial stages in lowering noise pollution and raising the standard of living for Barisal City Corporation citizens.

## Acronyms

DoE = Department of Environment, WHO = World Health Organization, EC = European Commission, U. S. (E. P. A) = United States (Environmental Protection Agency) GIS = Geographic Information System [18].

## Funding

No external funding is received for this article.

## Ethics approval and consent to participate

Not applicable.

## Availability of data and materials

All data generated or analyzed during this study are available for sharing when the appropriate request is directed to the corresponding author.

## CRediT authorship contribution statement

**Rifat Islam:** Research design, conceptualization, Investigation, Writing – original draft, Formal analysis. **Aliza Sultana:** Supervision, research design, Conceptualization, Validation, statistical analysis, Writing – review & editing manuscript. **Md. Selim Reja:** Data collection, Formal analysis, review manuscript. **Dr. Ashraf Ali Seddique:** Visualization, Validation, review manuscript. **Md. Rajib Hossain:** Visualization, Resources, review manuscript.

## Declaration of competing interest

The authors declare that they have no known competing financial interests or personal relationships that could have appeared to influence the work reported in this paper.

## References

[1] Stanchina M.L., Abu-Hijleh M., Chaudhry B.K., Carlisle C.C., Millman R.P. (2005). The influence of white noise on sleep in subjects exposed to ICU noise. Sleep.

[2] Andersen Z.J., Sram R.J., Ščasný M., Gurzau E.S., Fucic A., Gribaldo L., Rossner P., Rossnerova A., Kohlová M.B., Máca V., Zvěřinová I., Gajdosova D., Moshammer H., Rudnai P., Knudsen L.E. (2016). Newborns health in the Danube Region: environment, biomonitoring, interventions and economic benefits in a large prospective birth cohort study. Environ. Int..

[3] Kephalopoulos A. (2014). Road traffic noise and sleep quality in the urban environment. Sleep Med. Rev..

[4] Sultana A., Paul A.K., Nessa M.U. (2020). The status of noise pollution in the major traffic intersections of khulna city corporation city in Bangladesh and its possible effect on noise-exposed people. European Journal of Environment and Earth Sciences.

[5] Bunn W.H., Hallowell C.R., Hess J.E. (2016). Railway noise and sleep: a review of the literature. Sleep Med. Rev..

[6] Rahman M.M., Chowdhury M.A., Alam M.M. (2022). Noise pollution from construction activities in urban areas: levels, impacts, and mitigation strategies. Sustainable Environment Research.

[7] Uddin M.S., Al-Battoush A.M., Al-Mukhtar M.A., Shakil M.M. (2018). Assessment of noise pollution from miking activities in Dhaka city, Bangladesh. Journal of Environmental Public Health.

[8] Blomkvist V., Hygge S., Gustafsson P.O., Högberg L. (2005). Perceived noise and psychosocial stress at work: a longitudinal study. Occup. Environ. Med..

[9] Ighoroje A.D.A., Marchie C., Nwobodo E.D. (2004).

[10] Olaosun A.O., Ogundiran O., Tobih J.E. (2009). Health hazards of noise: a review article. Res. J. Med. Sci..

[11] Esmeray E., Eren S. (2021). GIS-based mapping and assessment of noise pollution in Safranbolu, Karabuk, Turkey. Environ. Dev. Sustain..

[12] Masum M.H., Pal S.K., Akhie A.A., Ruva I.J., Akter N., Nath S. (2021). Spatiotemporal monitoring and assessment of noise pollution in an urban setting. Environmental Challenges.

[13] Hossain M.S., Nahar N., Shaibur M.R., Bhuiyan M.T., Siddique A.B., Al Maruf A., Khan A.S. (2024). Hydro-chemical characteristics and groundwater quality evaluation in south-western region of Bangladesh: a GIS-based approach and multivariate analyses. Heliyon.

[14] Wei W., Van Renterghem T. (2016). Dynamic noise mapping based on noise measurement networks. Sci. Total Environ..

[15] Cai H., Wang Z., Sun D. (2015). Urban traffic noise mapping based on the integration of a genetic algorithm and a sound propagation model. Environ. Monit. Assess..

[16] Oyedepo S.O., Adeyemi G.A., Olawole O.C., Ohijeagbon O.I., Fagbemi O.K., Solomon R., Ongbali S.O., Babalola O.P., Dirisu J.O., Efemwenkiekie U.K., Adekeye T., Nwaokocha C.N. (2019). A GIS – based method for assessment and mapping of noise pollution in Ota metropolis, Nigeria. MethodsX.

[17] Olgun R., Karakuş N., Selim S. (2024). Assessment and mapping of noise pollution in recreation spaces using geostatistic method after COVID-19 lockdown in Turkey. Environ. Sci. Pollut. Res..

[18] Yang R., Xing B. (2021). A comparison of the performance of different interpolation methods in replicating rainfall magnitudes under different climatic conditions in Chongqing Province (China). Atmosphere.

[19] Van Renterghem T., Botteldooren D., Verheyen K. (2012). Road traffic noise shielding by vegetation belts of limited depth. J. Sound Vib..

[20] Sonaviya D., Tandel B. (2020). Integrated road traffic noise mapping in urban Indian context. Noise Mapp..

[21] Di H., Shi Y., He M., Wang Y., Now 16, have to change as 21 (2018). Traffic noise pollution assessment and its influence on residents' annoyance in urban areas: a case study of Shanghai, China. Environ. Sci. Pollut. Control Ser..

[22] Abbaspour K.C., Haghighi M., Moshtaghi M.J. (2015). A novel approach to traffic noise mapping and assessment in urban areas: a case study of Ahvaz, Iran. Environ. Monit. Assess..

[23] Lagonigro W.A., Ciancarella G., Fornaciari M. (2018). Traffic noise mapping and environmental exposure assessment: a case study of a southern Italian city. Environ. Monit. Assess..

[24] Olayinka O.S. (2013). Effective noise control measures and sustainable development in Nigeria. World Journal of Environmental Engineering.

[25] Pandya G.H. (2003). Assessment of traffic noise and its impact on the community. Int. J. Environ. Stud..

[26] Bari N.M., Bari M.N., Biswas A., Baki A.A. (2017). Determination of noise level of different places of Rajshahi city. Architecture and Civil Engineering.

[27] Nipa N., Seddique A.A., Hossain M., Amin A. (2022).

[28] Joonhee Lee, Lily M. (2020). Wang; Investigating multidimensional characteristics of noise signals with tones from building mechanical systems and their effects on annoyance. J. Acoust. Soc. Am..

[29] Bonani F., Ghione G., Pinto M.R., Smith R.K. (1998). An efficient approach to noise analysis through multidimensional physics-based models. IEEE Trans. Electron. Dev..

[30] Ato Armah F.K., Kwasi-Agyemang C., Agyemang-Duah W., Previous 25, now 30 (2010). The effect of environmental noise pollution on the health and well-being of residents in the Kumasi metropolis of Ghana. Environ. Health Perspect..

[31] Islam Rifat, Hossain Md Rajib (2023). Determining noise level situation. A Case Study of Lanchghat, Gopalganj.

[32] (2006). Noise Pollution Control Rules.

[33] Davis M.L., Cornwell D.A. (2008).

[34] Banerjee D., Chakraborty S.K., Bhattacharyya S., Gangopadhyay A. (2009). Appraisal and mapping the spatial-temporal distribution of urban road traffic noise. Int. J. Environ. Sci. Technol..

[35] Mawi R., Alam W., Nungate R. (2022). Article in Nature Environment and Pollution Technology.

[36] Farooqi Z.U.R., Nasir M.S., Nasir A., Zeeshan N., Ayub I., Rashid H., Qamar M.U., Sarwar A., Akram M.A. (2017). Evaluation and analysis of traffic noise in different zones of Faisalabad an industrial city of Pakistan. Geology, Ecology, and Landscapes.

[37] Karakus B.C., Yıldız S. (2020). Evaluation of noise pollution level from traffic for Siva's city using GIS-based noise indexes. Cumhuriyet Science Journal.

[38] Oguntunde P.E., Okagbue H.I., Oguntunde O.A., Odetunmibi O.O. (2019). A study of noise pollution measurements and possible effects on public health in Ota metropolis, Nigeria. Open Access Macedonian Journal of Medical Sciences.

[39] Rafael S.S., Fortes-Garrido J.C., Bolivar J.P. (2015). Characterization and evaluation of noise pollution in a tourist coastal town with an adjacent nature reserve. Appl. Acoust..

[40] Pelumi E., Oguntunde I. Hilary, Okagbue, Omoleye A., Oguntunde, Oluwole O., Odetunmibi (2019). A study of noise pollution measurements and possible effects on public health in ota metropolis, Nigeria. Open Access Macedonian Journal of Medical Sciences.

[41] Department of Environment, Bangladesh (DoE) (2023). Regulations for noise pollution. Under environmental quality sections.

[42] WHO (2011).

[43] United States Environmental Protection Agency (USEPA) (2024). Home page. https://www.epa.gov.

[44] Ali M.M., Ali M.L., Islam M.S., Rahman M.Z. (2016). Preliminary assessment of heavy metals in water and sediment of Karnaphuli River, Bangladesh. Environ. Nanotechnol. Monit. Manag..

[45] Aslam M., Khatun R., Ara R., Tama Z., Rahman M.M., Ali M.A. (2016). Effect of noise pollution on patients in hospitals and health clinics of Mymensingh sadar upazila. Int. J. Innovat. Appl. Stud..

[46] Hoque M.M.M., Basak L.K., Rokanuzzaman M., Roy S. (2013). Level of noise pollution at different locations in Tangail municipal area, Bangladesh. Bangladesh Journal of Science Research.

[47] Islam M.T., Nahar N., Islam M.J., Islam M.A., Hossen M.A.M. (2015). Traffic induced noise pollution and its impact on human health in chittagong city corporation. Journal of Environmental Science & Natural Resources.

[48] Jahan S., Munni S., Chandra Ghosh G. (2016). Nature environment and pollution Technology an international quarterly scientific journal noise pollution at major schools, colleges and hospitals in small urban area: focusing on jessore municipality. Bangladesh.

[49] Das S., Sultana S., Kabir M.M., Sultan M.A., Masum R. (2018). Assessment of noise pollution and its impact on human health in Dhaka Metropolitan City, Bangladesh. Int. J. Sustain. Dev..

[50] Allen R.W., Adar S.D. (2011). Are both air pollution and noise driving adverse cardiovascular health effects from motor vehicles?. Environ. Res..

[51] Belojevic G., Saric-Tanaskovic M. (2002). Prevalence of arterial hypertension and myocardial infarction in relation to subjective ratings of traffic noise exposure. Noise Health.

[52] Khaiwal R., Singh T., Tripathy J.P., Mor S., Munjal S., Patro B., Panda N. (2016). Assessment of noise pollution in and around a sensitive zone in North India and its non-auditory impacts. Sci. Total Environ..

[53] Peterson E.A., Augenstein J.S., Tanis D.C., Augenstein D.G. (1981). Noise raises blood pressure without impairing auditory sensitivity. Science.

[54] Ouis D. (1999). Exposure to nocturnal road traffic noise: sleep disturbance its after effects. Noise Health.

[55] Ato Armah F.K., Kwasi-Agyemang C., Agyemang-Duah W. (2010). The effect of environmental noise pollution on the health and well-being of residents in the Kumasi metropolis of Ghana. Environ. Health Perspect..

[56] Benfield J.A., Nurse G.A., Jakubowski R., Gibson A.W., Taff B.D., Newman P., Bell P.A. (2014). Testing noise in the field: a brief measure of individual noise sensitivity. Environ. Behav..

[57] Oyedepo S.O. (2012). Noise map: tool for abating noise pollution in urban areas. Sci. Rep..

[58] Oyedepo S.O. (2013). Development of noise map for Ilorin metropolis, Nigeria. Int. J. Environ. Stud..

[59] Ighoroje A.D.A., Marchie C., Nwobodo E.D. (2004). Noise-induced hearing impairment as an occupational risk factor among Nigerian traders. Journal of Physiological Science.

[60] Armah F.A., Obiri S., Yawson D.O., Pappoe A.N.M., Akoto B. (2010). Mining and heavy metal pollution: assessment of aquatic environments in Tarkwa (Ghana) using Multivariate Statistical Analysis. J Environ Stat.

[61] Goines L., Hagler L. (2007). Noise pollution: a modern plaque. South. Med. J..

[62] Benfield J.A., Nurse G.A., Jakubowski R. (2012). Testing noise in the field: a brief measure of individual noise sensitivity. Environ. Behav..

[63] Passchier-Vermeer W., Passchier W.F. (2000). Noise exposure and public health. Environ. Health Perspect..

[64] Yilmaz G., Hocanli Y. (2006). Mapping of noise by using GIS in Sanliurfa. Environ. Monit. Assess..

[65] Achilleos G.A. (2011). The inverse distance weighted interpolation method and error propagation mechanism – creating a DEM from an analogue topographical map. J. Spat. Sci..

[66] (2002). The European Parliament and the Council of the European Union, Directive 2002/49/EC of the European parliament and of the council of 25 June 2002 relating to the assessment and management of environmental noise, off. J. Eur. Commun..

